# Lipid composition of the Amazonian ‘Mountain Sacha Inchis’ including *Plukenetia carolis-vegae* Bussmann, Paniagua & C.Téllez

**DOI:** 10.1038/s41598-022-10404-8

**Published:** 2022-04-19

**Authors:** Nete Kodahl, Heidi Blok Frandsen, Henrik Lütken, Iben Lykke Petersen, Nelly Judith Paredes Andrade, Carmen García-Davila, Marten Sørensen

**Affiliations:** 1 Department of Plant and Environmental Sciences, University of Copenhagen, Thorvaldsensvej 40, 1871 Frederiksberg C, Denmark; 2 Department of Food Science, University of Copenhagen, 1958 Frederiksberg, Denmark; 3 Department of Plant and Environmental Sciences, University of Copenhagen, 2630 Taastrup, Denmark; 4 INIAP, Estación Experimental Central de La Amazonía, 170518 Orellana, Ecuador; 5 Instituto de Investigaciones de La Amazonía Peruana (IIAP), Loreto, Iquitos, Peru; 6 Present Address: SiccaDania, Pilehøj 18, 3460 Birkerød, Denmark

**Keywords:** Plant sciences, Plant domestication, Biodiversity, Nutrition, Lipids

## Abstract

Several Amazonian species of *Plukenetia* are remarkably rich sources of polyunsaturated fatty acids, in particular α-linolenic acid. The lipid composition of the large-seeded, recently described ‘Mountain Sacha Inchi’ *Plukenetia carolis-vegae* is reported here for the first time, and compared with *Plukenetia huayllabambana*, two cultivars of *Plukenetia volubilis*, and a newly developed hybrid between *P. volubilis* and *P. carolis-vegae*. All species and cultivars had a very high content of polyunsaturated fatty acids, 82.6–86.7% of total fatty acids, and at least 46.6% α-linolenic acid of total fatty acids. The highest content was found in *P. carolis-vegae* which had 57.4%. The exceptionally high α-linolenic acid content suggests that *P. carolis-vegae* may be an important plant-derived dietary source of this essential fatty acid and that the species has considerable potential for further domestication and commercialisation of its seeds and seed oil. A TAG analysis was carried out for the two *P. volubilis* cultivars, in which LLnLn and LnLL were most prevalent, and for *P. huayllabambana*, in which LLnLn constituted the largest fraction, followed by LnLnLn, indicating that this large-seeded species also has interesting dietary properties.

## Introduction

*Plukenetia carolis-vegae* Bussmann, Paniagua & C.Téllez is a recently discovered liana with large, edible, nut-like, oleaginous seeds^1^. Together with *Plukenetia huayllabambana* Bussmann, C.Téllez & A.Glenn and the new species *Plukenetia sylvestris* Card.-McTeag. & L.J.Gillespie it forms a high elevation species complex of ‘Mountain Sacha Inchi’, which are all native to the tropical regions of the Andes^2,3^. *Plukenetia carolis-vegae* and *P. huayllabambana* are two of five large-seeded species from the genus *Plukenetia* L. (Euphorbiaceae) which are known to have traditionally been cultivated for food and medicine, the remainder being *Plukenetia volubilis* L., ‘Sacha Inchi’ or ‘Inca Peanut’, and *Plukenetia polyadenia* Müll.Arg.*,* ‘Compadre-de-azeite’, which are both found in the Amazon Basin, and *Plukenetia conophora* Müll.Arg.*,* ‘Awusa’ or ‘African Walnut’, which is native to tropical central and west Africa^4^.

The genus *Plukenetia* has attracted increasing attention in recent years due to remarkably high amounts of polyunsaturated, essential fatty acids in the seeds of *P. volubilis*. However, although the seed biochemistry of *P. volubilis* is well established, only a few studies of *P. huayllabambana*, *P. polyadenia*, and *P. conophora* have been performed while no studies of the lipid composition of *P. carolis-vegae* exist. Nevertheless, the available data indicates that other large-seeded species of the genus have an oil composition that is similar to that of *P. volubilis* or perhaps even more nutritionally interesting.

**Figure 1:**
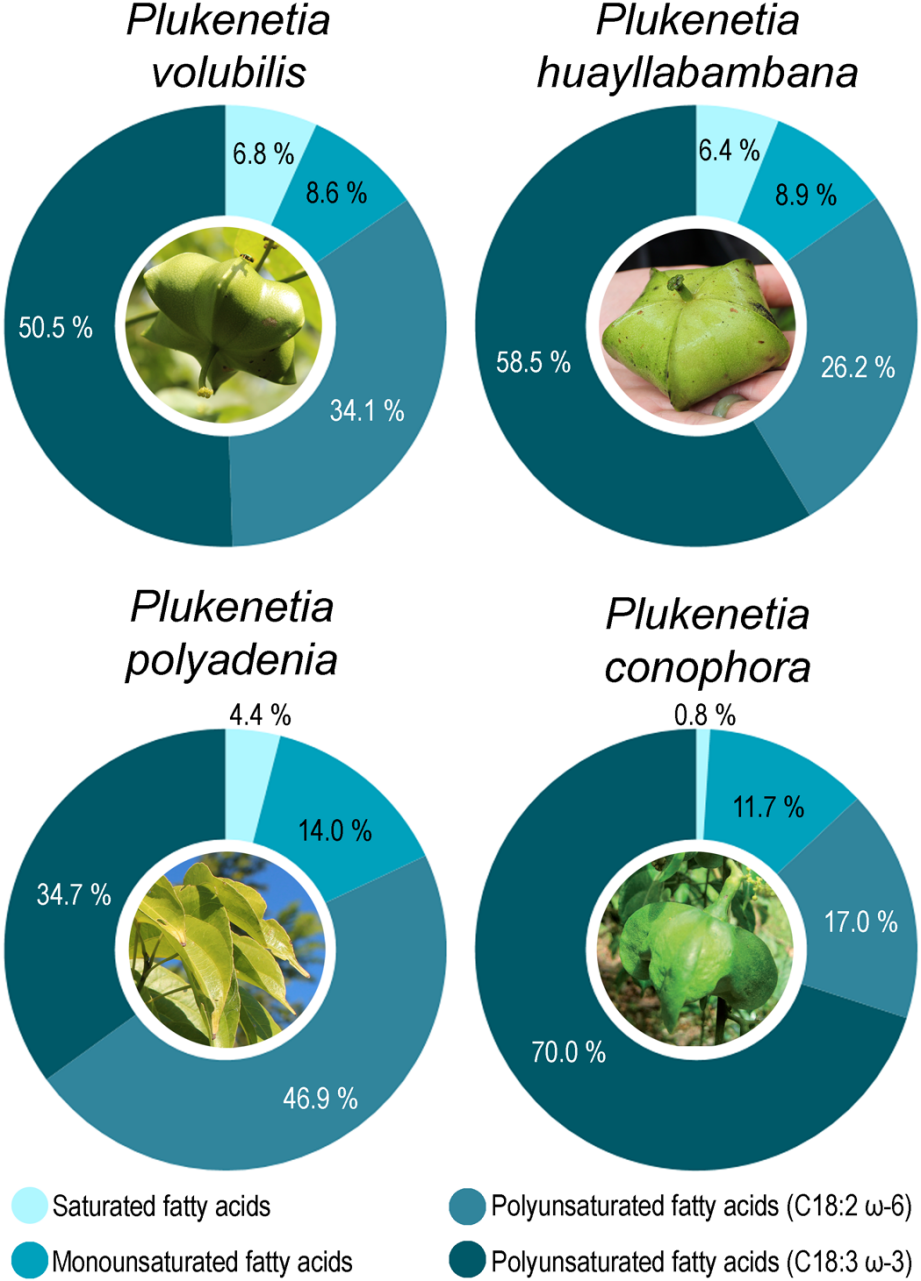
Comparison of the content of saturated, monounsaturated and polyunsaturated (including linoleic acid, C18:2 n-6, ω-6, and α-linolenic acid, C18:3 n-3, ω-3) fatty acids between four of the five large-seeded *Plukenetia* species known to have been cultivated. Data adapted from^7,14,15,18^. Image of *Plukenetia conophora* adapted from^13^.

The seeds of *P. volubilis* are known to have a lipid content of 33–58%, although the majority of studies report approx. 45–50%, of which approximately 77.5–84.4% are polyunsaturated fatty acids (PUFA), comprised of 35.2–50.8% α-linolenic acid (C18:3 n-3, ω-3, ALA) and 33.4–41.0% linoleic acid (C18:2 n-6, ω-6, LA). Of the remaining lipid fraction, 8.4–13.2% are monounsaturated fatty acids (MUFA), and 6.8–9.1% are saturated fatty acids (SFA) (Fig. 1;^5–12^). In comparison, the oil contents of the Neotropical species *P. huayllabambana* and *P. polyadenia* are similar to that of *P. volubilis* (approx. 49 and 47%, respectively) while the oil content of the Paleotropical *P. conophora* is somewhat greater (approx. 54%). The size of the PUFA fraction is comparable between the four species. However, both *P. huayllabambana* and *P. conophora* seed oil has a higher content of ALA than *P. volubilis* (approx. 58 and 70%, respectively), while that of *P. polyadenia* is lower (approx. 35%) (Fig. 1;^12,14–19^).

ALA is the precursor of eicosapentaenoic acid (EPA) and docosahexaenoic acid (DHA), which have numerous documented health effects, including prevention of cardiovascular disease, a protective effect in mood disorders, and improved cognitive function in children^20–22^. The inherent essentiality of ALA has been debated^23,24^, however, as more studies accumulate, the consensus has become that this plant-derived ω-3 fatty acid has a distinct role, and studies indicate that it influences brain health, vascular function, and the condition of skin and hair^23,24^. Further, several studies have shown benefits of ALA intake, e.g., the MRFIT (multiple risk factor intervention trial) study of 6250 men showed significant inverse associations between ALA and mortality from coronary heart disease, all cardiovascular diseases, and all-cause mortality^25^. Similarly, in the Lyon study—in which ALA content in the diet was a main difference between test and control diets—sudden death was reduced by 40% in the first year^24,26^. However, while the relevance of plant-derived ω-3 fatty acids is becoming clear, it may be complicated to incorporate larger amounts into the diet, and exploration of novel sources seems prudent.

*Plukenetia* seeds can be consumed whole, as ‘nuts’, or pressed for oil and the sensory acceptability of *P. volubilis* has been demonstrated to be good^27^. The high elevation ‘Mountain Sacha Inchi’ species complex, consisting of *P. carolis-vegae*, *P. huayllabambana*, and *P. sylvestris*, is sister to *P. volubilis* and all the species have larger seeds than *P. volubilis*^3,4^. The seeds of *P. carolis-vegae* are the largest in the group, and the available studies of the seed lipid composition of *P. huayllabambana* indicate that the ALA fraction of this species is greater than it is in the seed oil of *P. volubilis*^4,12,15–17^. Furthermore, a recent phylogenetic study suggests that *P. huayllabambana* is a hybrid between *P. volubilis* and *P. sylvestris*, the latter also being hypothesised to be the wild progenitor of *P. carolis-vegae*^3^. This relationship may show promise for a nutritionally interesting composition of the seed of *P. carolis-vegae*, and a study of the fatty acid composition of the seed oil of this species seems very relevant. Also, the current authors have demonstrated compatibility between *P. carolis-vegae* and *P. volubilis*, lending support to hybridisation events occurring within the species complex, and a study of the seed oil of the artificial hybrid *P. volubilis* × *P. carolis-vegae* may also aid in better understanding the nutritional qualities of the ‘Mountain Sacha Inchis’.

The current study aims to analyse the oil composition of *P. carolis-vegae* and *P. huayllabambana* using *P. volubilis* as a reference. Furthermore, the oil composition of the newly developed hybrid between *P. volubilis* and *P. carolis-vegae* will be studied. We hypothesise that *P. carolis-vegae* will have a similar lipid composition to *P. huayllabambana*, but with a higher fraction of ALA, and that *P. volubilis* × *P. carolis-vegae* will have a composition within the range of the parents’ lipid composition*.*

## Materials and methods

### Plant material

*Plukenetia volubilis* L. seeds were obtained from Joya de los Sachas, Orellana, Ecuador (‘*P. volubilis* Ecuador’, 0° 20′ 25.3″ S 76° 52′ 27.7″ W) and from Tarapoto, San Martín, Peru (‘*P. volubilis* Peru’, 6° 31′ 41.2″ S 76°17′ 57.2″ W). *Plukenetia huayllabambana* Bussmann, C.Tellez & A.Glenn. seeds were collected in Chachapoyas, Amazonas, Peru (6° 28′ 56.9″ S 77°21′ 53.5″ W), and *Plukenetia carolis-vegae* Bussmann, Paniagua & C.Téllez seeds in Rodríguez de Mendoza, Amazonas, Peru (6° 23′ 45.8″ S 77° 34′ 11.0″ W). Hybrid seeds (‘*P. volubilis* × *P. carolis-vegae’*) were obtained from a controlled hybridisation using pollen of *P. carolis-vegae* from Rodríguez de Mendoza, Amazonas, Peru (6° 23′ 45.8″ S 77° 34′ 11.0″ W), to fertilise a plant of *P. volubilis* from Pampamonte, San Martín, Peru (6° 21′ 23.5″ S 76° 35′ 38.8″ W). Seeds were collected at full maturity as evaluated by capsule colour and dehiscence to ensure comparable stages of seed lipid synthesis. In order to best compare species with different ecological niches, care was taken to collect seeds from vigorous plants at least 2 years of age grown under suitable conditions. All collections were carried out in May and early June and from each species or cultivar an estimated 10 g of seeds were collected for oil extraction. All methods were performed in accordance with the relevant guidelines and regulations and permissions for the collection of plant material were obtained. Voucher specimens of the plant material will be deposited at University of Copenhagen (C) during 2022.

### Oil extraction

The testae of the seeds was disrupted using a mortar and pestle and carefully removed, following which the seeds were milled using a coffee grinder.

Supercritical fluid extraction was performed on a laboratory scale unit (Spe-ed SFE, Applied Separations, Allentown, PA, U.S.A.) using 10 mL extraction tubes. Oil from milled seeds (2 g) was extracted in triplicates, with exception of the *P. volubilis* × *P. carolis-vegae* sample for which material was only available for two extractions. The samples were subjected to supercritical carbon dioxide (99.7% purity, Air Liquide SA, Taastrup, Denmark) for 30 min at 500 bar and 60 °C at a flow rate of 2.5 L min^−1^.

### Triacylglycerol analysis

The triacylglycerol (TAG) profile of the oils were analysed by enhanced liquid chromatography (EFLC) on a SFE/HPLC hybrid system (Agilent Infinity 1260, Santa Clara, U.S.A.) according to the method of Buskov et al.^28^. A BDS hypersil C18 column (250 × 4.6 mm, 5 µm particle size, Thermo Fisher Scientific) was used and isocratic elution was performed with acetonitrile:2-propanol:CO_2_ (56:14:30) at a flow rate of 1.5 mL min^−1^. The column temperature was set to 40 °C, and the column back pressure was 200 bar. The TAGs were detected with UV detection at 210 nm, and with ELSD (evaporative light scattering detection, Agilent 1900 Infinity, Santa Clara, U.S.A). The ELSD conditions were as follows: nebulizer temperature of 68 °C, evaporator temperature of 40 °C, and nitrogen pressure of 0.15 MPa. A 5 µL injection loop was used for analysing the TAG profile. For identification of the individual TAG composition preparative EFLC was applied in combination with GC-FAME analysis of each fraction. For the preparative EFLC a 200 µL injection loop was used and fractions collected every minute. Each fraction was then subjected to the FAME (fatty acid methyl esterification) procedure, and the fatty acid identified using a Supelco 37 Component FAME mix (Sigma-Aldrich, Denmark) and with spiking of individual fatty acid methyl ester standards (Sigma-Aldrich, Denmark).

### Fatty acid analysis

The FAME procedure was performed according to the AOAC Official Method 969.33^29^, with modifications as follows; 20 mg oil was added to 1 mL methanolic NaOH (0.5 M) with 200 µL internal standard (heptanoic acid, decanoic acid, and heptadecanoic acid; 10 mg/mL) (Sigma-Aldrich, Denmark), and vortex mixed with 1.5 mL boron-trifluoride methanol (BF_3_) solution. The mixture was heated at 70 °C for 2 min, where after a saturated NaCl solution was added and the FAMEs extracted into cyclohexane (1 mL).

The fatty acid profile was determined using gas chromatography (GC) with flame ionization detection (FID) using the Agilent Technologies GC System 7820A (Santa Clara, California, USA). A Supelco SP™-2380 capillary column (L × I.D. 30 m × 0.25 mm, d_f_ 0.20 μm) was used. Supelco 37 Component FAME mix (Sigma-Aldrich, Denmark) was used as a standard. The temperature program was as follows: initial temperature of 60 °C hold 2 min, ramp to 200 °C hold 0 min, and ramp to 240 °C hold 7 min. Helium was used as the carrier gas with a flow of 2.477 mL min^−1^, the injection volume was 1 mL with a split ratio of 52:1, and cyclohexane was used as a solvent.

The samples collected from preparative EFLC were evaporated to dryness before the esterification process, and a few variations were made to the procedures described above. Less solvents were used; 100 µL methanolic NaOH, 150 µL BF_3_ solution, and 200 µL cyclohexane. In the GC analysis, injection was changed to splitless, but otherwise no changes were made to the GC method.

### Data analysis

An analysis of variance (ANOVA) test followed by a Tukey HSD test was used to determine significant differences in the fatty acid composition of the different species and cultivars of *Plukenetia*.

## Results and discussion

### Fatty acid profile

#### Plukenetia volubilis

The fatty acid composition of *P. volubilis* is the most well studied in the genus, and the results from the two *P. volubilis* accessions from Ecuador and Peru in the current study are similar to previous results. The most abundant fatty acid in the seed oil of *P. volubilis* from Ecuador and Peru, respectively, is α-linolenic acid (C18:3 n-3, ω-3, ALA; 51.5 ± 3.3 and 46.6 ± 1.2%), followed by linoleic acid (C18:2 n-6, ω-6, LA; 32.5 ± 3.9 and 36.5 ± 0.8%), oleic acid (C18:1, OA; 8.5 ± 1,2 and 8.3 ± 0,4%) and smaller amounts (< 5%) of palmitic (C16:0), stearic (C18:0), eicosanoic (C20:0), and eicosenoic acids (C20:1; Fig. 2). Earlier studies have found approx. 35–51% ALA, 33–41% LA, and 8–11% OA in the seed oil of *P. volubilis*^5–12^. The absolute values vary between studies, but comparisons of different accessions or cultivars of *P. volubilis* within single studies also demonstrate a large amount of variability^5^. The seed oil of the Ecuadorian accession in the current study contains slightly more ALA and slightly less LA than previously observed in *P. volubilis*, however, as we observed small morphological differences between the accessions from Peru and Ecuador, we hypothesise that this may be attributed to genetic differences, although growing conditions including elevation and temperature may also have had an effect^30,31^.

**Figure 2:**
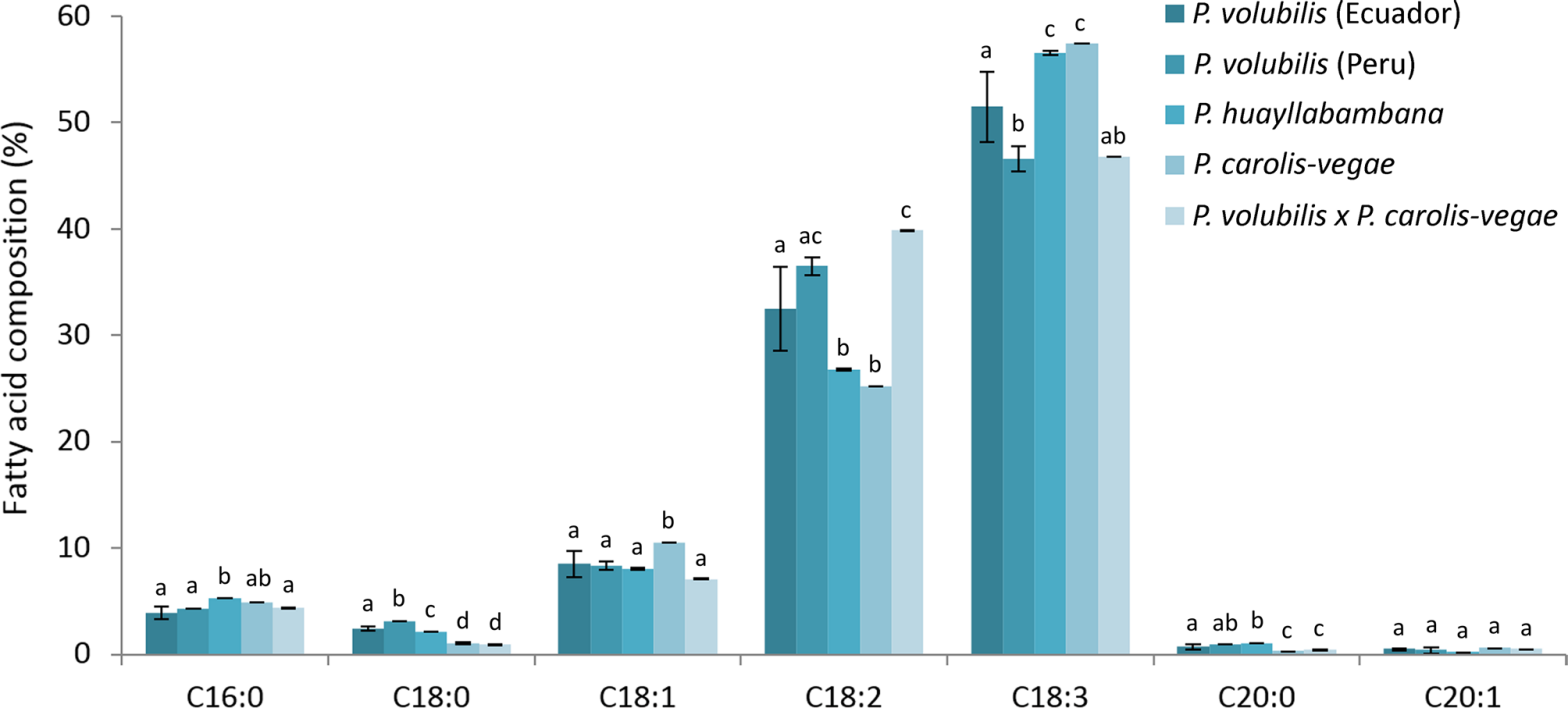
Composition of the lipid fraction of different *Plukenetia* species and cultivars, n = 3 (except *P. volubilis* × *P. carolis-vegae*, n = 2). C16:0, palmitic acid; C18:0, stearic acid; C18:1, oleic acid; C18:2, linoleic acid; C18:3, linolenic acid; C20:0, eicosanoic acid; C20:1, eicosenoic acid. Letters indicate significant differences.

Regardless of the compositional variation, the amount of ALA in the seed oil of *P. volubilis* is high, and only a few vegetable oils are comparable. Linseed (*Linum usitatissimum* L.) and chia (*Salvia hispanica* L.) are known for their high ALA content, and according to Ciftci et al.^32^, linseed oil contains 58.2 ± 0.64% ALA, 15.3 ± 1.01% LA, and 18.1 ± 0.45% OA, while chia oil contains 59.76 ± 0.13% ALA, 20.37 ± 0.19% LA, and 10.53 ± 0.17% OA. In comparison, the common cooking oils olive (*Olea europaea* L.) and sunflower (*Helianthus annuus* L.) contain < 1% ALA, while rapeseed oil (*Brassica napus* L.) contains approx. 10% ALA^33–35^.

#### Plukenetia huayllabambana

*Plukenetia huayllabambana* is one of the more recently described species of *Plukenetia*^2^ and have very large seeds; estimated as 6627 mm^3^ per seed compared with the approx. 997 mm^3^ of *P. volubilis*^4^. According to a recently published revised classification of the *Plukenetia* genus, *P. huayllabambana* is a putative hybrid between *P. volubilis* and the newly described *P. sylvestris*, a large-seeded species of the high elevation species complex sister to *P. volubilis*^3^. Our analysis shows that seed oil from *P. huayllabambana* has a significantly higher content of ALA (56.6 ± 0.2%) than *P. volubilis* from both Ecuador and Peru (51.5 ± 3.3 and 46.6 ± 1.2%, respectively), while the content of LA is also significantly lower (26.8 ± 0.1%, Fig. 2). This content corresponds well with the values previously reported for *P. huayllabambana*, which range from 51.3 to 58.2% ALA and from 25.8 to 29.3% LA; an ALA content generally exceeding that of *P. volubilis*^12,15,17^.

#### Plukenetia carolis-vegae

The oil composition of *P. carolis-vegae* was analysed for the first time in the current study. *Plukenetia carolis-vegae* has been hypothesised to be a cultivated and fully or semi-domesticated species derived from wild, naturally occurring populations of *P. sylvestris*. Further, *P. carolis-vegae* is a part of the high elevation ‘Mountain Sacha Inchi’ species complex sister to *P. volubilis* also consisting of *P. huayllabambana* and *P. sylvestris*^3^. The oil composition of *P. carolis-vegae* is of particular interest since the seeds are the largest in the species complex; approx. 7069 mm^3^ per seed^4^.

The seed oil of *P. carolis-vegae* was found to contain 57.4 ± 0.0% ALA (Fig. 2), which was the highest measured value in the study and was significantly different from the values of ALA measured in the seed oil of both *P. volubilis* cultivars and the hybrid *P. volubilis* × *P. carolis-vegae.* It was also higher than the value measured for *P. huayllabambana*, albeit not significantly. Conversely, the value of LA was the lowest measured, 25.2 ± 0.0% of the seed oil, which was significantly lower than the values measured in the oil of the *P. volubilis* cultivars and the hybrid, and also lower, though not significantly, than the value measured for *P. huayllabambana* seed oil. The very high ALA content of *P. carolis-vegae* may be caused by genetic factors or may be related to altitude and temperature; the material of *P. carolis-vegae* was collected at the highest altitude of the ‘Mountain Sacha Inchis’ in this study (1610 m), and Cai et al. (2012) observed that the ALA content of *P. volubilis* generally increased with higher altitude and decreasing temperatures^30^. However, studies of the oil composition of *P. carolis-vegae* cultivated at lower altitudes need to be conducted to assess whether the high ALA content is due to environment, genetics or both.

The amount of OA in *P. carolis-vegae* seed oil was 10.5 ± 0.0%, which was significantly higher than in both *P. volubilis* cultivars, *P. huayllabambana*, and the hybrid *P. volubilis* × *P. carolis-vegae*. The content of eicosenoic and palmitic acid in *P. carolis-vegae* seed oil (0.6 ± 0.0 and 4.9 ± 0.0%, respectively) was found to be mostly similar to the content in the seed oil of the other analysed species. In contrast, the content of eicosanoic and stearic acid (0.3 ± 0.0 and 1.0 ± 0.1%, respectively) was significantly lower than in both the *P. volubilis* cultivars and *P. huayllabambana* but similar to the levels in the hybrid. Overall, the most striking difference between the oils was the very high ALA content of *P. carolis-vegae* seed oil, a property which may be promising for further cultivation and domestication of the species.

#### *Plukenetia volubilis* × *Plukenetia carolis-vegae*

The ALA content of the *P. volubilis* × *P. carolis-vegae* hybrid seed oil is 46.8%, which is similar to that of the two *P. volubilis* cultivars, but lower than that of *P. huayllabambana* and *P. carolis-vegae*. Conversely, the LA content of the oil is 39.9%, which is significantly higher than all other samples except *P. volubilis* from Peru (Fig. 2). The OA lipid fraction is 7.1%, which is similar to that of the two *P. volubilis* cultivars and *P. huayllabambana*, but significantly different from that of *P. carolis-vegae*. Since the *P. volubilis* × *P. carolis-vegae* hybrid is a cross between *P. volubilis* and *P. carolis-vegae*, it is to be expected that fatty acid composition of the oil will be in between the composition of these two. While the values are not significantly different from those of *P. volubilis* from Peru, they are not very similar to the values of *P. carolis-vegae*, although the fruit morphology of the hybrid shows similarity with that of *P. carolis-vegae.* This dissimilarity may be due to either the genetic composition of this specific cross or may be an effect of environmental conditions. The hybrid was cultivated in a nursery at a lower altitude than the collection altitudes of either of the parents, and if altitude and temperature is indeed an essential driver of conversion from ALA to LA^30^, this might have influenced the seed oil composition of the hybrid.

### Triacylglycerol (TAG) profile

The distribution of fatty acids in the TAG molecules varies between species and cultivars and is responsible for the chemical, physical, and biological properties of oils and fats^36^. In *P. volubilis* from Ecuador, *P. volubilis* from Peru, and *P. huayllabambana*, 15 different TAGs were identified, containing six different fatty acids (Table 1).

**Table 1:** Percentages of different TAGs in Plukenetia species and cultivars, n = 3 (except *P. volubilis* × *P.carolis-vegae*, n = 2). E, eicosanoic acid (C20:0); L, linoleic acid (C18:2); Ln, α-linolenic acid (C18:3); O, oleic acid (C18:1); P, palmitic acid (C16:0); S, stearic acid (C18:0).

No	TAG	% per species		
		*P. volubilis* (Ecuador)	*P. volubilis* (Peru)	*P. huayllabambana*
1	LnLnLn	13.5 ± 1.3	12.5 ± 2.3	23.5 ± 0.8
2	LLnLn	35.0 ± 2.2	28.8 ± 2.5	35.6 ± 0.9
3	LnLL	21.5 ± 0.3	22.1 ± 1.8	14.1 ± 0.8
4	OLnLn	7.5 ± 0.1	6.4 ± 0.9	6.6 ± 1.0
5	PLnLn	2.4 ± 0.1	2.5 ± 0.6	4.6 ± 0.3
6	LLL	2.7 ± 0.6	3.8 ± 0.2	0.9 ± 0.0
7	OLLn	7.4 ± 0.6	8.4 ± 0.4	5.4 ± 0.4
8	PLLn	2.9 ± 0.4	3.9 ± 0.4	3.4 ± 0.3
9	SLLn	0.9 ± 0.2	1.4 ± 0.4	1.1 ± 0.3
10	OLL	1.1 ± 0.4	1.7 ± 0.2	0.7 ± 0.3
11	PSLn	1.8 ± 0.4	2.4 ± 0.3	1.7 ± 0.3
12	LPP	1.9 ± 0.2	3.9 ± 1.3	1.6 ± 0.3
13	EOL	0.4 ± 0.1	0.5 ± 0.1	0.3 ± 0.1
14	PSL	0.6 ± 0.2	1.2 ± 0.3	0.4 ± 0.2
15	EOO	0.3 ± 0.0	0.5 ± 0.2	0.2 ± 0.1

The most abundant TAG in the *P. volubilis* cultivars from Ecuador and Peru, and in *P. huayllabambana* was LLnLn, constituting 35.0 ± 2.2, 28.8 ± 2.5, and 35.6 ± 0.9%, respectively. However, a comparably very high amount of LnLnLn was found in *P. huayllabambana*; 23.5 ± 0. 8%. Following LLnLn, the predominant components in the two *P. volubilis* cultivars from Ecuador and Peru were LnLL (21.5 ± 0.3 and 22.1 ± 1.8%, respectively) and LnLnLn (13.5 ± 1.3 and 12.5 ± 2.3%, respectively), while in *P. huayllabambana* they were LnLnLn (23.5 ± 0.8%) and LnLL (14.1 ± 0.8%). In all samples, TAGs composed of polyunsaturated Ln (ALA) and L (LA) constituted more than two-thirds of the total TAG molecules (72.8% in *P, volubilis* from Ecuador, 67.2 in *P. volubilis* from Peru, and 74.1% in *P. huayllabambana*). Moreover, most of the identified TAGs (88. 5–95.9%) contained at least one residue of ALA.

These results correspond well with the TAG composition in *P. volubilis* oil measured by Fanali et al.^37^, who identified LLnLn as the most abundant TAG, and found that > 80% of TAGs contained ALA. The predominant TAGs after LLnLn in *P. volubilis* were LnLL and LnLnLn^37^. Similarly, Chasquibol et al.^15^ found LLnLn to be the most prevalent TAG in both *P. volubilis* and *P. huayllabambana*.

### Genetic control of the fatty acid composition

**Figure 3:**
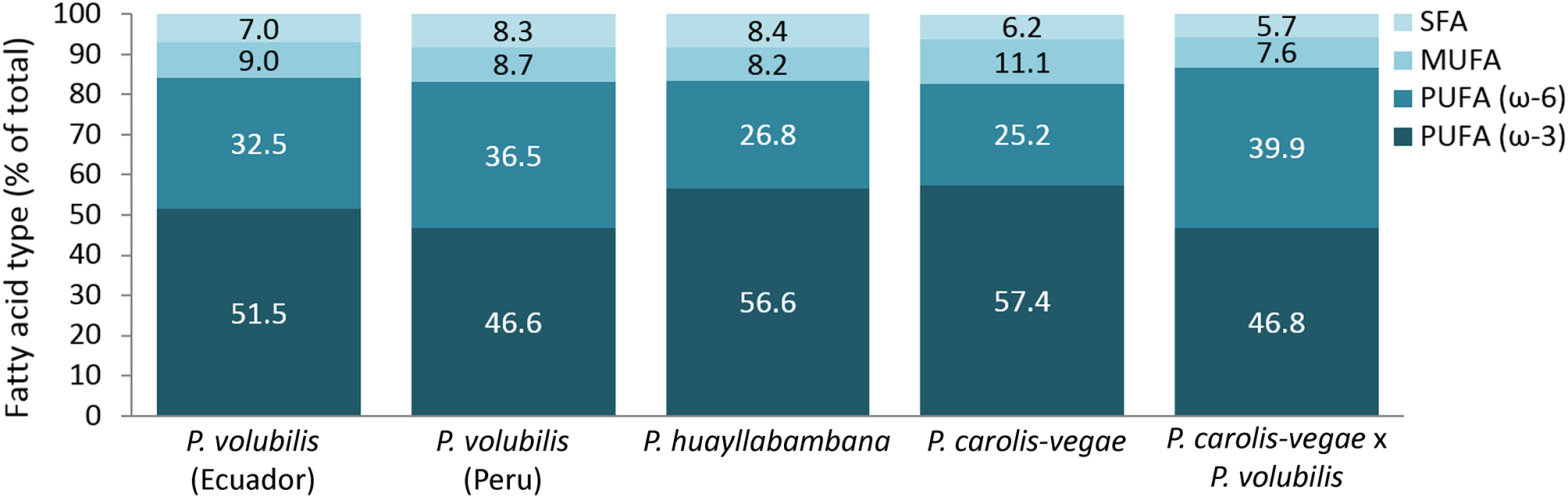
Relative distribution of saturated and unsaturated fatty acids in different species and cultivars of Plukenetia, n = 3 (except *P. volubilis* × *P. carolis-vegae*, n = 2). MUFA, monounsaturated fatty acids; PUFA, polyunsaturated fatty acids; SFA, saturated fatty acids.

Across all the examined species and cultivars, the SFA content is relatively low, ranging from 5.7% in *P. carolis-vegae* × *P. volubilis* to 8.4% in *P. huayllabambana.* Similarly, the MUFA content ranges from 7.6% in *P. carolis-vegae* × *P. volubilis* to 11.1% in *P. carolis-vegae* (Fig. 3). The remainder of the fatty acids is PUFA, comprised of ALA and LA, in total 84% of the seed oil in *P. volubilis* from Ecuador, 83.1% in *P. volubilis* from Peru, 83.4% in *P. huayllabambana*, 82.6% in *P. carolis-vegae*, and 86.7% in *P. carolis-vegae* × *P. volubilis*. Comparatively, linseed and chia oil contain approx. 74 and 80% PUFA, respectively^32^. The total PUFA content observed in the current study is largely similar across the species and cultivars. However, the relative fractions of ALA and LA vary considerably (Fig. 3), with *P. carolis-vegae* seed oil containing the highest amount of ALA (57.4%) and the *P. carolis-vegae* × *P. volubilis* hybrid containing the lowest (46.8%) although it has the highest total amount of PUFA. The differences in the composition of the PUFA fraction might be a result of genetic differences between the species and cultivars in combination with environmental factors.

The common pathway of PUFA biosynthesis in plants is initiated in the plastid with the formation of acyl-chains by the fatty acid synthase (FAS) complex, generating C16:0 and C18:0 fatty acids. Desaturation ensues by the action of a stearoyl-acyl carrier protein desaturase (SAD) to form OA (C18:1), which is further desaturated to LA (C18:2) by fatty acid desaturase-2 (FAD2) in the endoplasmic reticulum, and the third double bond is introduced at the ω-3 position of LA by fatty acid desaturase-3 (FAD3), also in the endoplasmic reticulum^38,39^. Until recently, the molecular mechanisms underlying the synthesis of the very high PUFA content in *P. volubilis* had not been elucidated, although a few studies had been published^31,40,41^. However, in a study by Yang et al.^39^ two *FAD* genes named *PvFAD2* and *PvFAD3* were isolated from *P. volubilis* and demonstrated to catalyse the synthesis of LA and ALA, respectively, although the authors point out that their results do not fully explain the massive accumulation of PUFA in *P. volubilis* seeds. Nevertheless, the differences observed between the species and cultivars included in the current study may at least in part be caused by differences in the expression of *PvFAD2* and *PvFAD3*. If so, *P. carolis-vegae* may have a relatively high expression of *PvFAD3*, leading to the synthesis of a very high amount of ALA in the seed oil, while, conversely, *P. volubilis* from Peru may have a lower expression of *PvFAD3*, yielding a lower amount of ALA in the seed oil. Furthermore, it can be speculated that the similar amounts of PUFA, but varying compositions of the PUFA fraction, observed in almost all the species and cultivars of the study could be an effect of LA being used as a substrate for PvFAD3 to produce ALA, reducing the amount of LA while increasing the amount of ALA.

Intriguingly, *P. carolis-vegae* × *P. volubilis* has the highest PUFA fraction of all the studied species and cultivars of *Plukenetia*, although the ALA fraction is comparably small. This characteristic could be caused by a higher expression of genes early in the biosynthetic pathway of ALA synthesis, e.g. *SAD*, in combination with a lower expression of *PvFAD3* compared to the other samples. However, it is also possible that a lower growing temperature would have induced activity of PvFAD3 and led to a higher accumulation of ALA relatively to LA; Yang et al.^39^ found that the activity of PvFAD3 was sensitive to temperature when expressed in yeast (*Saccharomyces cerevisiae*) cells, with low temperature (20 °C) significantly increasing biosynthesis of ALA. Accordingly, an analysis of the seed oil of all the ‘Mountain Sacha Inchis’, including the new species, *P. sylvestris*, and *P. volubilis*, grown at 20 and 30 °C might be useful in better understanding the very high accumulation of PUFA, primarily ALA in the large-seeded species of *Plukenetia*.

## Conclusion

The seed oil of the ‘Mountain Sacha Inchis’ *P. carolis-vegae* and *P. huayllabambana*, the two cultivars of *P. volubilis*, and the newly developed hybrid between *P. volubilis* and *P. carolis-vegae* contains exceptionally high amounts of PUFA, at least 82%, and a remarkably high amount of ALA (C18:3 n-3, ω-3), at least 46%. It is of particular interest that the very large-seeded *P. carolis-vegae* has the highest ALA content in the lipid fraction, > 57%, followed by *P. huayllabambana*, *P. volubilis*, and *P. volubilis* × *P. carolis-vegae*. This underlines that *P. carolis-vegae* is a highly promising underutilised crop that may provide many benefits for farmers as well as consumers.

*Plukenetia carolis-vegae*, but also *P. huayllabambana* and *P. volubilis*, has a huge potential for further domestication and industrialisation, due to the extraordinary nutritional qualities of the seed oil. Additionally, cultivation of the ‘Mountain Sacha Inchis’ may provide both nutritional and economic benefits for farmers and their communities. Finally, as demonstrated by the very high PUFA content of the *P. volubilis* × *P. carolis-vegae* hybrid, hybridisation breeding using ’Mountain Sacha Inchi’ germplasm might grant exciting opportunities.
